# Author Correction: Subpicosecond metamagnetic phase transition in FeRh driven by non-equilibrium electron dynamics

**DOI:** 10.1038/s41467-023-38263-5

**Published:** 2023-05-03

**Authors:** Federico Pressacco, Davide Sangalli, Vojtěch Uhlíř, Dmytro Kutnyakhov, Jon Ander Arregi, Steinn Ymir Agustsson, Günter Brenner, Harald Redlin, Michael Heber, Dmitry Vasilyev, Jure Demsar, Gerd Schönhense, Matteo Gatti, Andrea Marini, Wilfried Wurth, Fausto Sirotti

**Affiliations:** 1 The Hamburg Centre for Ultrafast Imaging, Hamburg University, Hamburg, Germany; 2 Deutsches Elektronen-Synchrotron DESY, Hamburg, Germany; 3 Istituto di Struttura della Materia—Consiglio Nazionale delle Ricerche (CNR-ISM), Division of Ultrafast Processes in Materials (FLASHit), Monterotondo Stazione, Italy; 4 European Theoretical Spectroscopy Facility (ETSF), https://www.etsf.eu/; 5 CEITEC BUT, Brno University of Technology, Brno, Czech Republic; 6 Institute of Physical Engineering, Brno University of Technology, Brno, Czech Republic; 7 Johannes Gutenberg-Universität, Institute of Physics, Mainz, Germany; 8 LSI, CNRS, CEA/DRF/IRAMIS, École Polytechnique, Institut Polytechnique de Paris, Palaiseau, France; 9 Synchrotron SOLEIL, L’Orme des Merisiers, Gif-sur-Yvette, France; 10 Physique de la Matiére Condensée, CNRS and École Polytechnique, IP Paris, Palaiseau, France

**Keywords:** Electronic properties and materials, Ferromagnetism, Phase transitions and critical phenomena

Correction to: *Nature Communications* 10.1038/s41467-021-25347-3, published online 24 August 2021

In the authors final submission, the Supplementary Information was uploaded as a ZIP file, containing a non-compliable TeX file, rather than in PDF or word document. The Supplementary movies referred to within the text were not included in the final version as part of the Supplementary Information ZIP file.

The attached files are the correct versions of the Supplementary Information and Supplementary movies referred to in the text, contained in the attached ZIP file and contain no changes.

## Supplementary information

Updated Supplementary Information

