# Supplementary material for: Subpicosecond metamagnetic phase transition in FeRh driven by non-equilibrium electron dynamics
Source: Nat Commun. 2021 Aug 24;12:5088. doi: 10.1038/s41467-021-25347-3 (PMC8384879; doi:10.1038/s41467-021-25347-3)
Supplement: Supplementary file 1 — Supplementary Information [file 41467_2021_25347_MOESM1_ESM.zip › Pressacco_ncomms_12_5088_2021_SI.pdf]

SUPPLEMENTARY INFORMATION

Subpicosecond metamagnetic phase transition in FeRh driven by non-equilibrium electron dynamics

Federico Pressacco[1,2,*], Davide Sangalli[3,4], Vojtěch Uhlíř[5,6], Dmytro Kutnyakhov[2], Jon Ander Arregi[5], Steinn Ymir Agustsson[7], Günter Brenner[2], Harald Redlin[2], Michael Heber[2], Dmitry Vasilyev[7], Jure Demsar[7], Gerd Schönhense[7], Matteo Gatti[8,4,9], Andrea Marini[3,4], Wilfried Wurth[1,2], and Fausto Sirotti[9,10]

[1]The Hamburg Centre for Ultrafast Imaging, Hamburg University, Luruper Chaussee 149, 22761, Hamburg, Germany
[2]DESY Photon Science, Hamburg Germany
[3]Istituto di Struttura della Materia—Consiglio Nazionale delle Ricerche (CNR-ISM), Division of Ultrafast Processes in Materials (FLASHit), Via Salaria Km 29.5, CP 10, I-00016 Monterotondo Stazione, Italy
[4]European Theoretical Spectroscopy Facility (ETSF)
[5]CEITEC BUT, Brno University of Technology, Purkyňova 123, 612 00 Brno, Czech Republic
[6]Institute of Physical Engineering, Brno University of Technology, Technická 2, 616 69 Brno, Czech Republic
[7]Johannes Gutenberg-Universität, Institute of Physics, Staudingerweg 7, 55128 Mainz, Germany
[8]LSI, CNRS, CEA/DRF/IRAMIS, École Polytechnique, Institut Polytechnique de Paris, F-91120 Palaiseau, France
[9]Synchrotron SOLEIL, L'Orme des Merisiers, Saint-Aubin, BP 48, F-91192 Gif-sur-Yvette, France
[10]Physique de la Matiére Condensée, CNRS and École Polytechnique, IP Paris, F-91128 Palaiseau, France
*e-mail: federico.pressacco@desy.de

A. Structural and magnetic characterization of FeRh films

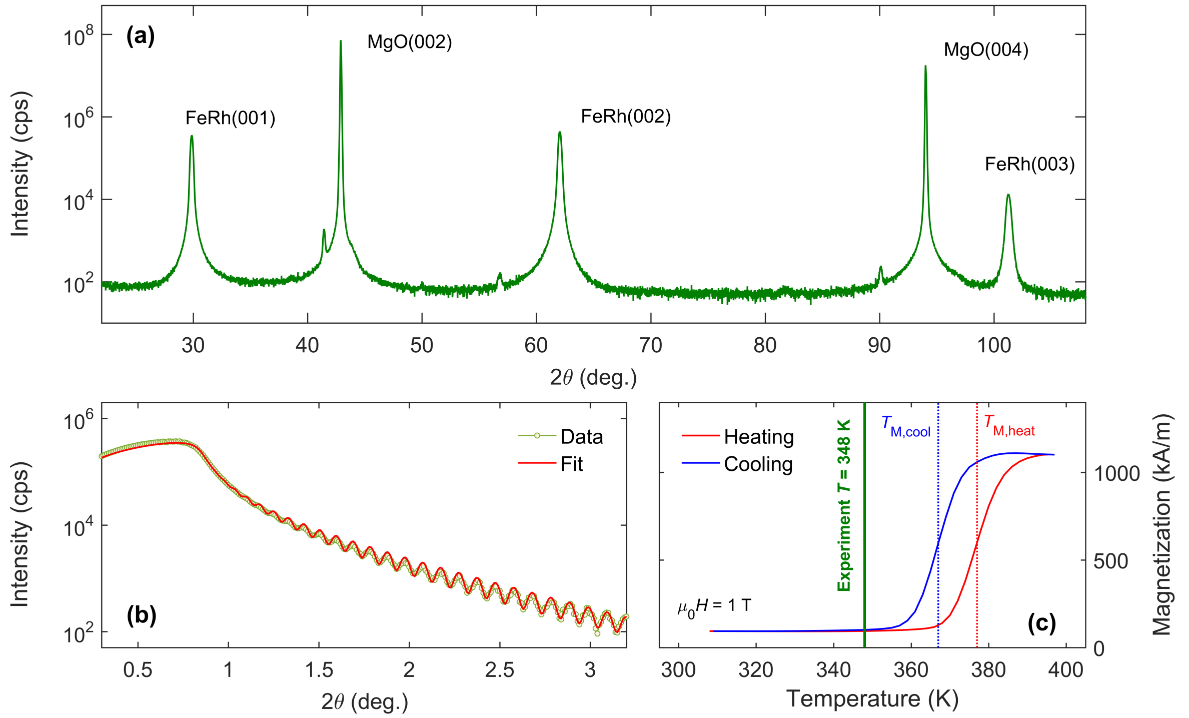

Figure S1. Structural and magnetic characterization of the 80-nm-thick FeRh film. **(a)** Room temperature θ-2θ x-ray diffraction scan ($\lambda = 1.5406$ Å), indicative of a highly textured FeRh(001) layer on top of a single-crystal MgO(001) substrate. The out-of-plane lattice parameter at 300 K is $c = 2.9887$ Å. **(b)** X-ray reflectivity measurement and fit (obtained using the `GenX` software[1]) revealing an FeRh film thickness of 81.6 ± 0.1 nm and roughness of 0.60 ± 0.02 nm. Additional structural parameters of the film (e.g., crystallographic grain size, in-plane epitaxy) are fully comparable to FeRh films grown under equivalent conditions and studied in our previous reports[2]. **(c)** Temperature-dependent magnetization data obtained via vibrating sample magnetometry under the application of an in-plane magnetic field of 1 T. The phase-transition temperatures during the heating and cooling cycle are are 377 K and 367 K, respectively (vertical dashed lines). The temperature span of the transition for the whole film during heating and cooling is 22 K and 16 K, respectively. Time-resolved photoelectron spectroscopy measurements were performed at 348 K (vertical solid line).

B. Photoelectron spectra measurements at different laser fluences

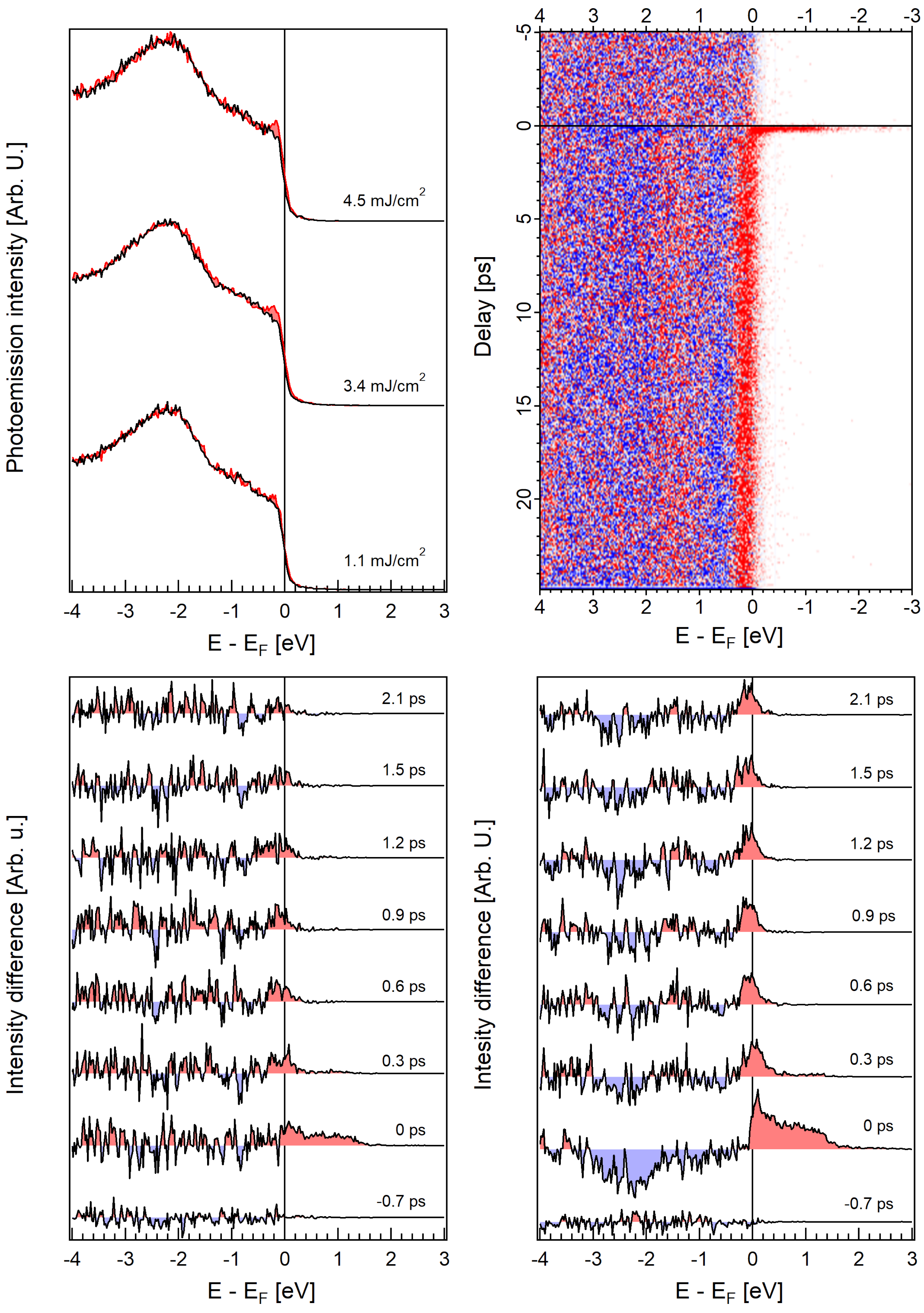

Figure S2. **(a)** Pump-probe photoemission intensity spectra obtained for different values of the laser-fluence (1.1, 3.4 and 4.5 mJ/cm^2). The spectra are integrated over a 1 ps delay interval before (blue lines) and after the laser pulse ($t = 2$ ps, color lines). The appearance of the peak-like minority band feature just below E_F, which is a fingerprint of the first-order phase transition, is present for laser fluences above ~ 3 mJ/cm^2 (for a base sample temperature of 348 K). **(b)** Photoemission intensity difference map measured with a laser fluence of 4.5 mJ/cm^2 on a 28 ps delay interval, demonstrating the persistence of the FM phase fingerprint for time delays $t > 20$ ps. **(c,d)** Selected difference photoemission spectra (represented as in Figure 2b of the manuscript) obtained for laser fluences of 1.1 mJ/cm^2 and 4.5 mJ/cm^2, respectively.

C. Simulated charge and spin density dynamics

We provide four animations showing the simulated dynamics of charge density variations $n(\mathbf{r},t) - n^{eq}(\mathbf{r})$ as represented in panels **a** and **b** of Fig. 5 of the main manuscript during two time-windows. The first time window (-65 fs $< t < -55$ fs) corresponds to the beginning of the photoexcitation process and shows the laser-induced charge oscillations for about four periods of the main laser frequency (for $\lambda = 800\,\mathrm{nm}$, $T = 2.67\,\mathrm{fs}$). During this time window, $n(\mathbf{r},t) - n^{eq}(\mathbf{r}) \sim 10^{-7}$ a.u. (see **Supplementary Videos 1** & **2**). The second time window (135 fs $< t <$ 137.5 fs) is close to the end of the optical pulse where a persistent change of the charge density is observed. This variation is due to the update of the mean field felt by the electrons, which redistribute accordingly in the unit cell. Laser-induced charge oscillations are still present, but only constitute a correction on top of the much larger persistent variation. At the end of the laser pulse, $n(\mathbf{r},t) - n^{eq}(\mathbf{r}) \sim 10^{-5}$ a.u. (see **Supplementary Videos 3** & **4**.)

Additional references

1. Björck, M. & Andersson, G. *GenX*: an extensible X-ray reflectivity refinement program utilizing differential evolution. *Journal of Applied Crystallography* **40**, 1174–1178 (2007). URL https://doi.org/10.1107/S0021889807045086.

2. Arregi, J. A., Caha, O. & Uhlíř, V. Evolution of strain across the magnetostructural phase transition in epitaxial FeRh films on different substrates. *Phys. Rev. B* **101**, 174413 (2020). URL https://doi.org/10.1103/PhysRevB.101.174413.
